# Habitual Alcohol Consumption and Metabolic Syndrome in Patients with Sleep Disordered Breathing

**DOI:** 10.1371/journal.pone.0161276

**Published:** 2016-08-18

**Authors:** Su Jung Choi, Sung Ik Lee, Eun Yeon Joo

**Affiliations:** 1 Department of Neurology, Neuroscience Center, Samsung Medical Center, Sungkyunkwan University School of Medicine, Seoul, Korea; 2 Department of Nursing, Samsung Medical Center, Department of Clinical Nursing Science, Graduate School of Clinical Nursing Science, Sungkyunkwan University, Seoul, Korea; 3 Department of Health Sciences and Technology, SAIHST, Sungkyunkwan University, Seoul, Korea; 4 Department of Neurology, Inam NeuroScience Research Center, Sanbon Hospital, Wonkwang University School of Medicine, Gunpo-si, Korea; National Taiwan University College of Medicine, TAIWAN

## Abstract

To investigate the associations between amount of habitual alcohol consumption (HAC) and prevalence of metabolic syndrome (MetS), sleep, and sleep-disordered breathing (SDB). We enrolled 683 untreated SDB male patients (age: 54.4 ± 7.80 y, apnea-hypopnea index (AHI): 29.0 ± 21.53/h). HAC was assessed as the average number of drinks consumed per week during the past 12 months. Anthropometric and biochemical markers were used to diagnose MetS. Clinical data and MetS components were compared according to the reported amounts of HAC (no drinking, light drinking <13, heavy drinking ≥13 drinks/week). As reported, 78.9% of the participants (n = 539) were regular drinkers; 33.7% (n = 230) were habitually heavy drinkers (mean: 30.7 drinks/week), and 45.2% (n = 309) were light drinkers (5.1 drinks/week). The overall prevalence of MetS was 36.9% (n = 252) and was most common in heavy drinkers (40.5%). Compared to non-drinkers and light drinkers, heavy drinkers had the greatest body mass index (BMI) and waist circumference. Central obesity, hypertension, and hyperglycemia were most prevalent in heavy drinkers. Sleep quality and severity of SDB were the worst in heavy drinkers. After adjusting for age, AHI, and BMI, heavy drinkers had a 1.71 times greater risk of MetS when compared with non-drinkers, and light and heavy drinkers had a 2.06 and 2.11 times higher risk of severe SDB than non-drinkers. HAC may increase the prevalence of MetS and deteriorate sleep in relation to amount of alcohol intake. Even light drinkers had more than twice higher risk of severe SDB than non-drinkers.

## Introduction

The relationships among habitual alcohol consumption (HAC), sleep-disordered breathing (SDB), and metabolic syndrome (MetS) are complex. There is no doubt that the prevalence rate of MetS is increased in people with SDB, and vice versa [1–3] because the two diseases share similar risk factors, such as obesity [1,4,5]. There have been inconsistent reports concerning the association between alcohol consumption and MetS [6–9]. This relationship may be different according to HAC amount or type of alcoholic beverage consumed [6,7,9,10]. Recently, continuous and moderate to heavy drinking in particular were both reported to be associated with MetS in Korean men [11]. However, a meta-analysis demonstrated that alcohol consumption <40 g/day in men and <20 g/d in women actually lowered the risk of MetS [12]. While mild to moderate alcohol consumption has a favorable influence on lipid metabolism, abdominal obesity, and glucose regulation [13], alcohol consumption also causes hypertension [14] and hypertriglyceridemia [15], factors of alcohol-related MetS. This controversy could be related to the complex mechanistic relations between alcohol consumption and each component of MetS [12]. Beverage-specific effects may also play a role. In contrast, wine and beer have been associated with a lower prevalence of MetS [7].

The effects of alcohol on SDB may vary according to the timing of alcohol intake and HAC amount. Most studies in which defined quantities of alcohol were administered just prior to sleep have shown detrimental effects on respiration, including increasingly frequent and longer duration of hypopnea and apnea events [16–22]. Alcohol intake in large quantities (equivalent to a blood concentration >0.075 g/dl) significantly aggravated SDB [17,23], while the negative effect of lower doses (0.5–1.0 alcohol kg/body weight) on SDB is less clear [19]. Based on previous reports, it is reasonable to counsel patients with SDB or who are at risk for SDB not to drink alcohol immediately before bed. However, the cumulative effect of alcohol consumption on MetS and SDB has not been extensively investigated. A positive association between HAC amount and risk of SDB was reported in some studies [24,25]. Nevertheless, subsequent studies failed to duplicate the negative effects of alcohol consumption on SDB [26–28].

Despite a low incidence of obesity in Korea, the overall prevalence of SDB (apnea-hypopnea index, AHI ≥5/h) was 27% and 16% in men and women, respectively; these results were similar to those found in Western countries [29]. South Korea is one of the heaviest drinking nations in the world. On average, 85% of men and 44% of women consume more than two drinks/day, while 47% of men and 13% of women reported binge drinking ≥1 time/week [30]. Considering the variation in alcohol-attributable injuries according to race/ethnicity [31], the relatively high prevalence of MetS and SDB in Korea HAC may be attributable to HAC.

To the best of our knowledge, no previous study has investigated the relationship between HAC and MetS or severity of SDB. In this study, we aim to 1) compare the prevalence of MetS and sleep quality according to amount of HAC in male patients with SDB and 2) explore the relationships between HAC and MetS or severity of SDB.

## Methods

### Participants

This was a retrospective, cross-sectional study of participants who underwent night polysomnography (PSG) for diagnosis of SDB and screening tests as part of a health checkup program at the Health Promotion Center of Samsung Medical Center in Seoul, South Korea, from March 2007 to December 2014. Each participant underwent PSG within an average of 99.9±30.35 days following the health checkup tests. The study population included 818 consecutive SDB participants (AHI ≥ 5/h) who had undergone both examinations. All are male. We excluded participants with the following characteristics: age <20 or >75 y (n = 58), repeated examination (n = 47), and insufficient data (n = 30). The final analysis included 683 participants.

This study protocol was reviewed and approved by the Institutional Review Board at Samsung Medical Center. The requirement of informed consent was waived as we used non-identifiable data routinely collected during the health screening process.

### Methods and Variables

#### Definition of heavy drinking

HAC was assessed using questions about average weekly consumption during the past 12 months. The respondents were categorized as non-drinkers, light drinkers, and heavy drinkers. Heavy drinking is defined as a quantity of alcohol consumption that exceeds an established threshold value [32]. Individuals who drink in excess of these guidelines are thought to be at increased risk for certain adverse health events. The National Institute of Alcohol Abuse and Alcoholism sets this threshold at more than 14 standard drinks per week for men (or >4 drinks per occasion) in the United States (US) [33]. A “drink” was defined as the alcohol quantity contained in a standard cup used for specific alcoholic beverages: 30 ml of liquor and 200 ml of beer (10 g of pure alcohol). However, Asians, including Koreans, lack the liver enzymes alcohol dehydrogenase and aldehyde alcohol dehydrogenase and experience more physiological dependency and social problems from only a small amount of alcohol [34,35]. We defined heavy drinking as >13 drinks per week and light drinking as <13 drinks per week in the current study [35]. Data of habitual alcohol consumptions of subjects can be found in S1 Table.

#### Health examination

We used self-administered health questionnaires, vital signs, anthropometric data, and venous blood samples obtained during a health examination. The health questionnaires assessed smoking, alcohol consumption, and history of hypertension, diabetes, and hyperlipidemia. Blood pressure was measured using a standardized sphygmomanometer after the participant had rested for at least five minutes. Height and weight were also obtained, and the body mass index (BMI) was calculated by dividing the weight (kg) by the square of height (m). Waist circumference was measured at the midpoint between the inferior margin of the last rib and the superior iliac crest. Venous blood samples were collected from each participant in the morning after an overnight fast of at least 8 h.

#### Metabolic syndrome

Based on the Joint Scientific Statement [36], MetS requires evidence of central obesity with two or more abnormal criteria of the following four components: hypertriglyceridemia, low high-density lipoprotein (HDL)-cholesterol, hypertension, and hyperglycemia. Central obesity was present in Asian males who had a waist circumference 90 cm or more. Hypertriglyceridemia was defined as an elevated triglyceride (TG) level ≥150 mg/dl or specific treatment for dyslipidemia. Low HDL-cholesterol was confirmed when the value was <40 mg/dl. Hypertension was defined as systolic blood pressure (SBP) ≥130 mmHg, diastolic blood pressure (DBP) ≥85 mmHg, or current antihypertensive treatment for previously diagnosed hypertension. Hyperglycemia was present when the participant exhibited a fasting serum glucose ≥100 mg/dl or had been previously diagnosed with diabetes mellitus.

#### Overnight polysomnography

Participants were instructed not to drink alcohol during the seven days before the sleep study, including the study night. Sleep studies were performed using a Remlogic (Embla Systems, Denver, CO, US). The recording time of the PSG lasted from approximately 22:00 on the night of the study to 07:00 the next day. Sleep architecture was scored in 30-sec epochs. The sleep stage and the scoring of respiratory events were performed according to the American Academy of Sleep Medicine Manual [37]. Apnea was defined when the duration of the ≥ 90% drop in sensor signal was at least 10 seconds. Hypopnea was the duration of the ≥ 30% drop in signal excursions for at least 10 seconds with ≥ 3% oxygen desaturation from pre-event baseline or the event was associated with an arousal.

### Statistical analyses

All statistical analyses were conducted using the Statistical Package for Social Sciences (SPSS) for Windows, version 20.0 (SPSS, Chicago, IL, US). The alpha level for statistical significance was set at *p* <0.05. Descriptive statistics of mean±SD and number (percentage) are summarized in Table 1. Normally distributed continuous data were analyzed using one-way analysis of variance (ANOVA), while skewed data were evaluated using the Kruskal-Wallis test. Categorical variables were analyzed using the Chi-square test. To examine the association between HAC and severe SDB after adjusting for possible confounders (age and BMI), univariate and multiple logistic regression analyses were conducted [38,39]. The results of the logistic regression analysis were reported as adjusted odds ratio (OR) with 95% confidence interval (CI). We also performed a multiple logistic regression analysis to examine the association between HAC and MetS after adjusting for possible confounders (age, AHI, and BMI). To evaluate the effect of both HAC and SDB on development of MetS, interaction analysis was performed using multiple regression.

**Table 1:** Characteristics of subjects with sleep disordered breathing according to habitual alcohol consumption.

	Non-drinkers (N = 144)	Light-drinkers (N = 309)	Heavy-drinkers (N = 230)	*p*
Age*, years	56.5±8.19	54.2±7.95	53.4±7.11	0.001
Smoking				
● Never smoking	75(52.1)	126(40.8)	84(36.5)	0.034
● Ex-smoker	44(30.0)	100(32.4)	78(33.9)	
● Current smoking	26(18.1)	83(26.9)	68(29.6)	
Alcohol consumption, /week	0	5.1±3.88	30.7±17.29	<0.001
Frequency of alcohol intake, /week	0	1.4±1.02	3.3±1.47	
● <1/month		7(2.3)	0(0)	<0.001
● 1/month		18(5.8)	0(0)	
● 2-4/month		215(69.6)	32(13.9)	
● 2-3/week		63(20.4)	132(57.4)	
● ≥4/week		6(1.9)	66(28.7)	
Average intake/case	0	3.9±2.47	10.4±5.72	
● <1 drink		14(4.5)	0(0)	<0.001
● 1–2 drinks		94(30.4)	0(0)	
● 3–4 drinks		93(30.1)	2(2.2)	
● 5–6 drinks		18(5.8)	8(3.5)	
● ≥7 drinks		90(29.1)	217(94.3)	
SBP, mmHg	118.6±15.53	119.4±14.39	121.3±14.74	0.185
DBP, mmHg	75.4±9.90	76.5±9.70	79.2±10.12	<0.001
**Anthropometric measures**				
● Waist circumference, cm	90.6±9.09	89.9±7.18	91.7±7.92	0.038
● Body mass index, kg/m^2^	25.9±3.76	25.4±2.73	26.1±3.07	0.026
**Biochemical markers**				
● Total cholesterol, mg/dL	188.9±35.96	189.1±40.28	193.4±35.63	0.226
● LDL*, mg/dL	119.6±32.83	121.4±31.15	120.2±31.50	0.834
● HDL, mg/dL	47.9±10.74	50.4±12.88	51.2±12.22	0.036
● Triglyceride, mg/dL	141.8±92.48	142.8±84.51	163.5±111.23	0.028
● AST	25.9±10.27	26.7±15.62	29.1±19.77	0.119
● ALT	30.5±16.82	32.5±32.94	32.5±30.02	0.767
● GGT	39.1±33.38	43.1±38.82	61.0±47.76	<0.001
● Fibrinogen, mg/dL	279.7±64.61	299.2±64.35	291.2±56.52	0.008
● Glucose, mg/dL	100.8±22.47	100.8±20.08	104.7±17.13	0.050
● HbA1c, %	5.8±0.91	5.7±0.76	5.8±0.73	0.304
● C-peptide, ng/mL	2.2±1.31	2.2±0.99	2.5±1.00	0.019
● Insulin, uIU/mL	8.9±4.68	8.7±5.01	9.4±5.66	0.335
**Metabolic syndrome**	50(34.7)	100(32.4)	102(44.3)	0.014
● Central obesity**	70(48.6)	159(51.5)	139(60.4)	0.043
● Higher TG***	77(53.5)	174(56.3)	139(60.4)	0.387
● HDL<40mg/dL	29(20.1)	65(21.0)	42(18.3)	0.725
● Hypertension^†^	80(55.6)	194(62.8)	157(68.3)	0.046
● Hyperglycemia^††^	66(45.8)	137(44.3)	128(55.7)	0.026

* ANOVA test

** Waist circumference ≥90cm

*** Triglyceride ≥150mg/dL or taking medication for dyslipidemia

^†^ SBP≥130mmHg or DBP≥85mmHg or taking medication,

^††^ Fasting serum glucose≥100mg/dL or DM history or taking medication

SBP; systolic blood pressure, DBP; diastolic blood pressure, LDL; low-density lipoprotein, HDL; high-density lipoprotein, AST; aspartate transaminase, ALT; alanine transaminase, GGT; gamma glutamyl tranferase, HbA1c; hemoglobin

## Results

All subjects were males and the mean age of the participants was 54.4 years, and their ages ranged from 20–74 years. The mean BMI was 25.8±3.10 kg/m^2^, while the average waist circumference was 90.7±7.89 cm. In all, 78.9% (539/683) reported drinking habits, and 33.7% (230/683) were classified as habitual heavy drinkers (≥13 drinks per week). Heavy drinkers consumed a weekly average of 30.7±17.29 drinks. Heavy drinkers were also the youngest (*F* = 7.24, *p* = 0.001) and had the highest BMI (*F* = 3.65, *p* = 0.026) and the largest waist circumference (*F* = 3.28, *p* = 0.038) compared to non-drinkers and light drinkers. Among biochemical markers, serum TG, aspartate transaminase (AST), gamma glutamic transferase (GGT), fibrinogen, glucose, and C-peptide level differed according to HAC amount. The prevalence of MetS in participants with SDB was 36.9% and was high in heavy drinkers (44.3%, *X*^*2*^ = 8.51, *p* = 0.014). Table 1 displays the participant characteristics and history of alcohol intake according to HAC.

**Fig 1:**
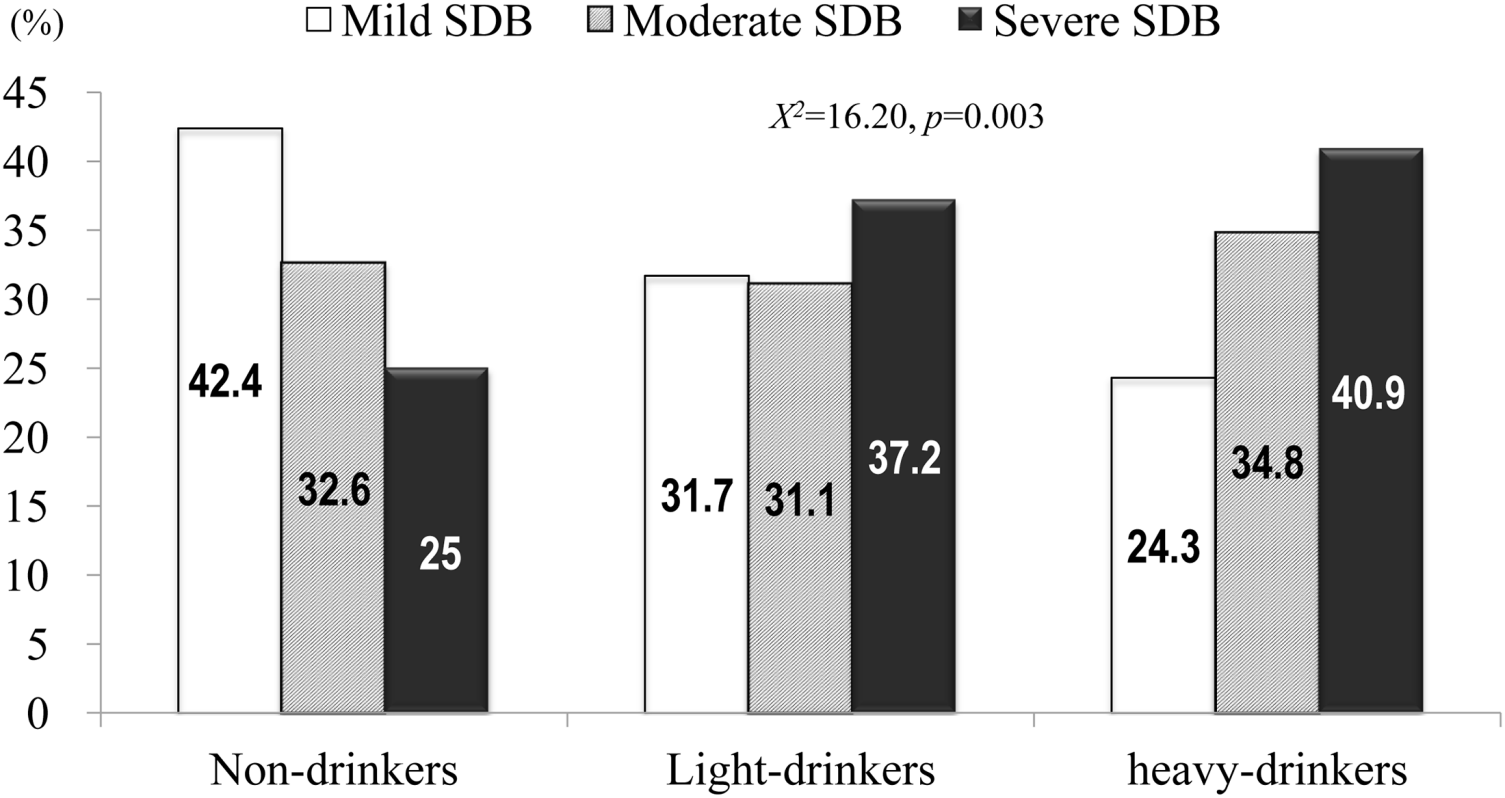
Proportion of sleep-disordered breathing (SDB) severity according to habitual alcohol consumption (HAC). The distribution of severity of SDB was significantly different according to HAC amount (*X*^*2*^ = 16.20, *p* = 0.003). The numbers of patients with severe SDB (apnea-hypopnea index ≥ 30/h) were the highest in heavy-drinkers. On the contrary, proportion of mild SDB was the greatest in non-drinkers.

The distribution of SDB severity was significantly different according to HAC amount (*X*^*2*^ = 16.20, *p* = 0.003) (Fig 1). The number of participants with severe SDB (AHI ≥ 30/h) was highest in the heavy drinkers. Sleep parameters obtained from overnight PSG were compared according to HAC and are summarized in Table 2. Severity of SDB, AHI, and sleep quality were the worst in heavy drinkers. Sleep structure was fragmented, and sleep efficiency was reduced in all participants, with no significant differences according to HAC amount.

**Table 2:** Polysomnography-based sleep parameters according to habitual alcohol consumption.

	Non-drinkers (N = 144)	Light-drinkers (N = 309)	Heavy-drinkers (N = 230)	*p*
TST, min	325.5±77.37	334.4±71.08	329.9±70.31	0.452
SOL, min	10.0±12.38	8.7±10.65	9.7±20.29	0.595
REM latency*, min	98.6±56.01	96.3±53.41	96.0±64.92	0.906
WASO,%	15.9±10.16	15.2±10.24	15.2±9.07	0.733
Sleep efficiency,%	81.7±11.11	82.9±10.79	83.0±10.01	0.448
**Sleep stage, %**				
● N1 sleep	24.2±11.71	24.8±12.42	27.3±12.82	0.025
● N2 sleep	55.1±11.13	54.4±11.72	51.9±11.16	0.012
● N3 sleep	2.5±4.38	2.3±3.99	1.8±3.70	0.218
● REM sleep	18.1±6.93	18.4±6.99	18.9±7.04	0.543
AHI, events/h	25.5±24.31	28.2±20.19	32.2±21.07	0.009
Nadir SaO_2_	82.8±7.35	82.3±6.68	81.1±7.44	0.045
Arousal index, events/h	30.1±13.75	31.7±15.09	34.5±17.03	0.019
● RAI	17.3±16.33	20.4±16.88	23.1±18.20	0.007
● MAI	1.4±3.77	1.2±3.08	0.9±2.22	0.220
PLM index, events/h	10.2±22.92	11.9±26.56	8.8±16.55	0.291

* ***ANOVA test*,** TST; total sleep time, SOL; sleep onset latency, WASO; wake after sleep onset, REM; rapid eye movement, AHI; apnea-hypopnea index, SaO_2;_ Oxygen saturation, RAI; respiratory arousal index, MAI; movement arousal index, PLM; periodic leg movement.

After adjusting for age, BMI, and AHI, the univariate and multiple logistic regression analyses revealed that heavy drinkers were more likely to have MetS than non-drinkers [odds ratio (OR) = 1.71 (1.01–2.88)] (Table 3). The relationship between HAC and SDB severity is presented in Table 4. After adjusting for age and BMI, heavy drinkers [OR = 2.11 (1.30–3.44)] and light drinkers [OR = 2.06 (95% CI = 1.29–3.31)] were more likely to have severe SDB than were non-drinkers. The interaction effect of HAC and SDB severity was not significant.

**Table 3:** Odds ratio (95% Confidence Intervals) for metabolic syndrome according to habitual alcohol consumption.

	Unadjusted	Adjusted for age	Adjusted for age, and AHI	Adjusted for age, AHI, and BMI
Non-drinkers	1.0 (reference)	1.0 (reference)	1.0 (reference)	1.0 (reference)
Light-drinkers	0.90(0.59–1.37)	0.91(0.60–1.34)	0.87(0.57–1.34)	1.13(0.68–1.88)
Heavy-drinkers	1.50(0.97–2.30)	1.52(0.98–2.35)	1.38(0.88–2.15)	1.71(1.01–2.88)

AHI; apnea-hypopnea index, BMI; body mass index

**Table 4:** Odds ratio (95% Confidence Intervals) for severe apnea-hypopnea index according to habitual alcohol consumption.

	Unadjusted	Adjusted for age	Adjusted for age, and BMI
Non-drinkers	1.0(reference)	1.0(reference)	1.0(reference)
Light-drinkers	1.78(1.14–2.77)	1.71(1.10–2.67)	2.06(1.29–3.31)
Heavy-drinkers	2.07(1.31–3.28)	1.97(1.24–3.16)	2.11(1.30–3.44)

BMI; body mass index

## Discussion

We observed the overall prevalence of MetS in Korean men with SDB to be 36.8%, and this value increased to 45.7% in heavy drinkers. In 2005, a longitudinal survey announced the incidence of MetS in Korean men as 10% [11]. A subsequent study in 2007 reported the prevalence of MetS as 31.3%, which was similar to the 34.2% reported by a US survey conducted from 1999–2006 [5]. The current study is noteworthy in that it is the most recent Asian data and includes a distinctive group with SDB. Accordingly, MetS is rapidly increasing in prevalence in Asian countries, including South Korea. Due to the rising incidence of high-fat diet and low physical activity in these areas, increasing abdominal circumferences and dyslipidemia may be contributing to the rapid growth of MetS in Asia [40]. In addition, we also focused on the effects of habitual alcohol consumption on the increase in MetS and aggravation of sleep quality and underlying SDB.

### Does alcohol consumption increase MetS?

Studies of the association between HAC and prevalence of MetS have reported conflicting data. A meta-analysis suggested that responsible alcohol intake (< 40 g/day in men and < 20 g/day in women) appears to be associated with a reduced prevalence of MetS [12]. However, a prospective observation study in Korea found that light (<14.9 g/day), moderate (15.0–29.9 g/day), and heavy drinkers (>30.0 g/day) had increased ORs of 1.51, 1.71 and 2.11, respectively, for developing MetS compared with non-drinkers [11]. Studies conducted in Korean people, including the current study, have not identified a positive effect of alcohol on MetS. In addition, standards of light or heavy drinking may vary greatly across studies and according to race or gender [41,42]. In the current study, we defined heavy drinkers as those who consumed more than 13 drinks (= 130 g) per week due to the lower alcoholysis ability of Asians [35]. Nonetheless, Korean men were still found to drink frequently. Over two-thirds of our participants drank habitually, and one-third of them were heavy drinkers. Contrary to Western reports, alcohol consumption has been shown to increase the prevalence of MetS in a Korean population. After adjusting for age, AHI, and BMI, we found that light drinkers had a 1.13 times higher risk of MetS and heavy drinkers (≥13 drinks per week) had a 1.71 times higher compared with non-drinkers. The risk of habitual heavy drinking in MetS was greater than that of age (OR: 1.04) or SDB severity (OR: 1.00) and was similar to that of BMI (OR: 1.72). We also noted that waist circumference and BMI were highest in heavy drinkers. As a result, the rate of MetS (44.3%) and the presence of at least half of the components of MetS (central obesity: 60.4%, hypertension: 68.3%, and hyperglycemia: 55.7%) were highest in heavy drinkers compared to non-drinkers or light drinkers. These findings suggest that heavy drinking increases waist and body weight as well as the prevalence of MetS.

In addition to HAC amount and components of MetS, we also suggest a crucial role of smoking on the high prevalence of MetS in patients with SDB. Nicotine is known to promote development of atherosclerosis by changing the lipid metabolism [43]. The higher smoking rates in light (26.9%) and heavy drinkers (29.6%) compared to non-drinkers (18.1%) in the current study might hinder the positive effect of alcohol on MetS. A beverage-specific effect also seems to be important. In the case of wine and beer, mild to moderate alcohol consumption was associated with a lower prevalence of MetS [7]. Almost all participants in Korean studies, including the current one, drink Soju, a highly distilled ethanol liquor made from sweet potatoes and tapioca and mixed with water, flavoring, and sweetener. Since Soju does not contain any other components, such as resveratrol or other antioxidants, it may have a lower preventive effect against MetS [44]. The possible differential effects among the different types of alcoholic beverages should be confirmed in a multiethnic population.

### Does habitual alcohol consumption aggravate SDB and sleep quality?

Alcohol intake immediately before sleep onset, even only a small amount, may induce or aggravate sleep-related respiratory events via reduced upper airway muscle tone and decreased sensitivity for oxygen desaturation during the events [19,21]. Yet, the effects of HAC on sleep quality or on the severity of SDB have been inconsistent [24–28]. We observed that the distribution of SDB severity was significantly different according to HAC amount. The proportion of mild SDB (AHI 5–15/h) was the highest (42.4%) in non-drinkers, while that of severe SDB was the greatest (40.9%) in heavy drinkers than in those with non-drinkers or light-drinkers. This result suggests that greater alcohol intake is associated with more severe SDB. The current study demonstrated that sleep quality deteriorated based upon HAC amount. Compared to non-drinkers or light drinkers, heavy drinkers had the most severe SDB (the highest AHI (32.2/h) and the lowest nadir oxygen desaturation (81.1%)) and the most fragmented sleep structures (the highest arousal indices (34.5/h) and N1 sleep proportion (27.3%)). Considering that the prevalence of SDB tends to increase with age [45], it is unusual that the heavy drinkers in the current study who experienced the most severe SDB were the youngest (mean age: 53.4 y). Therefore, we suggest the critical role of a heavy alcohol consumption habit on SDB because both light and heavy drinkers were significantly associated with more severe SDB compared to non-drinkers (ORs: 2.06 and 2.11, respectively). The estimated risks of HAC on SDB in the current study appear to be higher than in previous reports. In a cross-sectional study conducted in the US in 2011, participants who were both current and past alcohol consumers showed greater odds of having SDB (ORs = 1.52 and 1.65, respectively) than non-drinkers (Pan *et al*., 2014). However, in that same study, alcohol consumption status and sleep apnea were assessed using only yes-or-no questions [46]. This lack of objective assessment in sleep or alcohol intake could have limited the interpretation of the results.

A population-based study using pulse oximetry revealed that the ORs of current drinkers with an oxygen desaturation index ≥5/h were 1.19–1.95 [24]. It has been argued that the oxygen desaturation index obtained using pulse oximetry represents the severity of SDB, e.g., AHI. That study reported a positive association between an average alcohol intake and severity of SDB among Japanese men, which was similar to the current study. Additionally, they identified a stronger association between alcohol intake and SDB among non-overweight participants with SDB than overweight ones, which is contrary to the current study. Further evaluation is needed to determine whether SDB is related to racial differences, a beverage-specific effect, or method of assessment of alcohol intake.

Another population-based study from the US assessed SDB severity using overnight PSG [25]. In that study, men who consumed more alcohol had 25% greater odds of mild or worse SDB (OR = 1.25) for each increment of one drink per day, and women did not show any association between alcohol consumption and increased risk of SDB [25]. In the current study, we enrolled only men with SDB because there were few women with SDB without insomnia comorbidity at our hospital (<5% of the total). A lack of understanding about SDB in women in Korea (i.e., they seldom visit sleep clinics with SDB-related symptoms) is likely responsible for the deviated gender ratio of patients at our hospital.

The methodological limitations and strengths of our study need to be considered when interpreting our findings. First, this study was cross-sectional; thus, the results should not be interpreted as having a causal association due to a lack of temporality. Second, the study population was biased for any generalization of the results. The participants were all men who had been diagnosed with SDB. Moreover, most participants were white-collar workers from the middle socio-economic class. Subscribing to Confucian principles, traditional Korean society has allowed drinking for men, but not for women. As a society has changed, contemporary women drink at a younger age and consume larger amounts of alcohol than their prior generations [47]. Those young women do not usually take a health checkup examination or polysomnography in Korea. It incurs extremely biased gender proportion in this study. Third, a beverage-specific effect of alcohol may exist. Measurement of drinking was based on only one kind of beverage: Soju. We admit that a possible liquor-specific effect may exist in MetS and SDB. However, we assessed SDB severity and sleep quality using standard methods, obtained a detailed history of habitual alcohol consumption, and examined anthropometric measures and biochemical markers for accuracy.

In summary, this is the first study to explore the risk of HAC on MetS and sleep in male patients with SDB. HAC may increase the prevalence of MetS and deteriorate sleep quality in relation to HAC amount. Heavy drinkers are at higher risk of having MetS than non-drinkers or light drinkers. Nevertheless, even in light amounts, HAC may have a twice greater risk of severe respiratory-related disturbances during sleep in participants with SDB.

## Supporting Information

S1 Table Demographics of subjects. Demographics, habitual alcohol consumption, apnea-hypopnea index, and metabolic syndrome of subjects. Further explanation can be fond in the abbreviation datasheet included.
(XLS)
